# Adherence to clinical practice guidelines for Management of epithelial precancerous conditions and lesions in the stomach in Europe

**DOI:** 10.1055/a-2695-1376

**Published:** 2025-10-06

**Authors:** Filipa Fontes, Noa E. A. Kapteijn, Cesare Hassan, Charlene Deane, Margarida Cristiano, Henrique Fernandes-Mendes, Irina Luzko, Maša Čavlina Sevo, Orlaith Kelly, Gianluca Esposito, I Lisanne Holster, Michiel P. J. van der Horst, Charlotte F. Kweldam, Andrei Voiosu, Ricardo Marcos Pinto, Nuno Almeida, Colm O'Morain, Leticia Moreira, Jan Bornschein, Manon C.W. Spaander, Mário Dinis-Ribeiro

**Affiliations:** 1 Precancerous Lesions and Early Cancer Management Group, Research Center of IPO Porto (CI‐IPOP)/CI‐IPOP@RISE (Health Research Group), Portuguese Institute of Oncology of Porto (IPO Porto)/Porto Comprehensive Cancer Center (Porto.CCC), Porto, Portugal; 2 26706 Public Health and Forensic Sciences, and Medical Education Department, Faculty of Medicine, University of Porto, Porto, Portugal; 3 6993 Gastroenterology and Hepatology, Erasmus MC University Medical Center Rotterdam, Rotterdam, Netherlands; 4 9268 Digestive Endoscopy Unit, IRCCS Humanitas Research Hospital, Milan, Italy; 5 437807 Biomedical Sciences, Humanitas University, Milan, Italy; 6 Gastroenerology, Beacon Hospital Research Institute, Dublin, Ireland; 7 58867 Gastroenterology, Connolly Hospital Blanchardstown, Dublin, Ireland; 8 8863 Department of Medicine, Royal College of Surgeons in Ireland, Dublin, Ireland; 9 Gastroenterology, ULS de Coimbra, Coimbra, Portugal; 10 674892 Gastroenterology, Centro Hospitalar Universitário de Santo António, ULS de Santo António, Porto, Portugal; 11 16493 Gastroenterology, Hospital Clinic de Barcelona, Barcelona, Spain; 12 Institut d’Investigacions Biomèdiques August Pi i Sunyer (IDIBAPS), Centro de Investigación Biomédica en Red de Enfermedades Hepáticas y Digestivas (CIBEREHD), Barcelona, Spain; 13 112594 Endoscopy, Gastroenterology and Hepatology, University Hospital Centre Zagreb, Zagreb, Croatia; 14 58867 Gastroenerology, Connolly Hospital Blanchardstown, Dublin, Ireland; 15 117698 Medical-Surgical Sciences and Translational Medicine, Sant'Andrea Hospital, Rome, Italy; 16 7000 Gastroenterology, Maasstad Hospital, Rotterdam, Netherlands; 17 7000 Pathology, Maasstad Hospital, Rotterdam, Netherlands; 18 Gastroenterology, Colentina Clinical Hospital, Bucharest, Romania; 19 Gastroenterology, Tallaght Hospital/Trinity College Dublin, Dublin, United States; 20 Gastroenterology, Hospital Clínic de Barcelona, Barcelona, Spain; 21 11269 MRC Translational Immune Discovery Unit, John Radcliffe Hospital, Oxford, United Kingdom of Great Britain and Northern Ireland; 22 6396 Translational Gastroenterology and Liver Unit, Nuffield Department of Medicine, University of Oxford, Oxford, United Kingdom of Great Britain and Northern Ireland; 23 59035 Gastroenterology, Instituto Português de Oncologia do Porto, Porto, Portugal

## Abstract

**Background:**

The first international guideline for managing preneoplastic conditions of the stomach (MAPS I) was published in 2012, followed by an update (MAPS II) in 2019. As adherence to these guidelines remains uncertain, we evaluated adherence by comparing the management of preneoplastic gastric conditions before and after the introduction of MAPS I and II in selected European centers.

**Methods:**

Patient data were retrieved from nine endoscopy units in seven European countries during three periods: pre-MAPS I (2010/2011), post-MAPS I (2017/2018), and post-MAPS II (2022/2023). Screening and dyspepsia-related endoscopies were included. Data on endoscopies, histopathology, *Helicobacter pylori* treatment, and surveillance were collected using a standardized form. Adherence to nine MAPS recommendations was assessed, with improvement defined as a ≥10-percentage point increase in guideline-concordant management.

**Results:**

A total of 2426 patients were included. Over the years, most centers (57%) improved in seven of the nine recommendations. Virtual chromoendoscopy use improved in six centers, with four reporting its use in >50% of cases. All centers improved in performing biopsies from the antrum and corpus, five conducted random biopsies in nearly all patients, and four performed these plus a biopsy from the incisura in >90% of cases. Endoscopic scores for patient stratification were rarely used, although five centers improved in histological scoring or intestinal metaplasia subtyping. *H. pylori* treatment recommendations remained high (71%–100%), and endoscopic surveillance adherence improved in 4/7 centers.

**Conclusions:**

Adherence to MAPS guidelines improved in most centers; however, gaps in virtual chromoendoscopy, targeted biopsies, and endoscopic/histopathological scoring remain, potentially affecting surveillance recommendations. This underscores the need for a more tailored approach to enhance implementation.

## Graphical Abstract

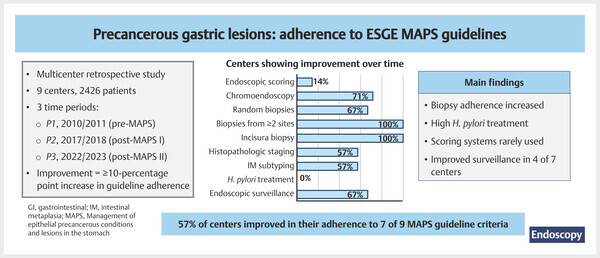

## Introduction

According to GLOBOCAN 2022 estimates, gastric cancer ranks as the fifth most frequently diagnosed cancer type and the fifth leading cause of cancer-related mortality, accounting for 4.9% of all cancer cases and 6.8% of cancer-related deaths worldwide 1 . In addition, gastric cancer is usually diagnosed at advanced stages of the disease, resulting in a 5-year relative survival rate ranging between 11.9% and 34.5% in Europe 2 . Process measures, such as adherence to guideline-recommended biopsy protocols and surveillance intervals, are essential indicators of quality of care and directly enhance the likelihood of detecting gastric neoplastic conditions at an earlier stage 3 4 . For example, evidence suggests that systematic adherence to biopsy protocols significantly increases the diagnostic yield of preneoplastic conditions, enabling timely interventions that may reduce gastric cancer incidence and mortality 5 6 .

Recognizing the importance of standardized practices to improve early detection and management of preneoplastic conditions of the stomach, a collaboration among leading European societies led to the publication of the first international guideline on this topic in 2012, entitled “Management of epithelial precancerous conditions and lesions in the stomach (MAPS)” 7 . This guideline, endorsed by the European Society of Gastrointestinal Endoscopy (ESGE), the Sociedade Portuguesa de Endoscopia Digestiva (SPED), the European Helicobacter and Microbiota Study Group (EHMSG), and the European Society of Pathology (ESP), focused on the assessment and management of atrophic gastritis, gastric intestinal metaplasia (GIM), and dysplasia. An updated version, MAPS II, was published in 2019 6 , highlighting the ongoing importance of a risk-stratified approach to the management of these premalignant conditions.

Despite efforts to promote adherence, research on guideline implementation is limited, with only a few large-scale studies addressing variations in adherence and identifying the barriers clinicians face in following these guidelines 8 9 10 11 . Moreover, the number of studies focusing on the factors influencing adherence, particularly across different healthcare settings and countries, remains limited. For instance, a 2012 survey by two Italian national gastroenterology societies reported that, while 69% of pathology reports included separate descriptions of biopsies from the antrum and corpus, the Sydney system was applied in only one-third of cases 8 . A subsequent 2018 survey found that approximately 90% of the Italian endoscopists followed the MAPS biopsy protocols for diagnosing and staging atrophic gastritis and GIM 9 . A retrospective study conducted in The Netherlands and the UK found that adequate surveillance was achieved in only 54.3% of patients diagnosed with GIM or gastric atrophy 10 . In the USA, a study of patients with newly diagnosed GIM showed limited adherence to management recommendations from both the American Gastroenterological Association (AGA) and ESGE. For instance, in only 42.3% of patients was *Helicobacter pylori* testing recommended after GIM was detected, just 22.0% had separate antral and corporal biopsies, and only 2.0% had recommendations on the interval for endoscopic surveillance documented in their records 11 .

Assessing adherence to the MAPS guidelines is crucial, as nonadherence may lead to suboptimal patient outcomes, including delayed diagnosis and missed opportunities for early intervention for gastric neoplasia. Additionally, variability in compliance across different countries and healthcare systems underscores the need for a comprehensive evaluation to identify potential barriers and improve clinical practice globally.

This study aimed to assess the current level of adherence to MAPS guidelines across European settings, focusing on variations in adherence between different countries and healthcare centers. By identifying gaps and variations, this research seeks to provide insights that can lead to targeted interventions to improve guideline implementation and, ultimately, patient care.

## Methods

### Study population and design

A total of 21 gastroenterology units from different countries across Europe were invited to take part in this study via an email invitation. Of these units, five declined the invitation and seven did not respond despite multiple follow-up attempts. Ultimately, nine centers agreed to participate, resulting in data collection from seven countries (Spain, The Netherlands, UK, Ireland, Italy, Portugal, and Romania) ( Table 1 ).

**Table:** **Table 1** Numbers of patients included in the analysis for each center and time period.

Center	Maximum number of patients contributed ^1^			
	All periods	2010/2011	2017/2018	2022/2023
Ospedale Universitario Sant'Andrea, Rome, Italy	400	100	100	100
Connolly Hospital Blanchardstown, Dublin, Ireland	200	–	100	100
Unidade Local de Saúde de Coimbra, Coimbra, Portugal	150	50	50	50
Unidade Local de Saúde de Santo António, Porto, Portugal	300	100	100	100
Colentina Clinical Hospital, Bucharest, Romania	200	–	100	100
Hospital Clínic de Barcelona, Barcelona, Spain	300	100	100	100
John Radcliffe Hospital, Oxford, UK	376	33	128	215
Maasstad Hospital, Rotterdam, The Netherlands	300	100	100	100
Erasmus Medical Center, Rotterdam, The Netherlands	300	100	100	100
All centers	2426	583	878	965
^1^ For some secondary analyses, the sample size may be smaller owing to additional inclusion criteria.				

The study included consecutive patients who had an upper gastrointestinal (GI) endoscopy for screening or investigation of dyspepsia during three time periods: 2010–2011 (pre-MAPS I guideline publication), 2017–2018 (post-MAPS I), and 2022–2023 (post-MAPS II). Patients with a prior diagnosis of gastric cancer, a history of gastric surgery, or known genetic cancer syndromes were excluded. Each participating center was asked to collect data from 100 consecutive patients for each specified period, to minimize selection bias and ensure representative sampling within the local clinical workflow.

### Data collection

Endoscopy, histopathology, and data related to patient treatment and surveillance were obtained from medical records using a standardized form, ensuring uniform data collection across all centers. Data from each setting were reported, aggregated, and anonymized at an individual level.

The outcomes reported by each center were: (1) proportion of patients in whom virtual chromoendoscopy was used; (2) proportion of patients in whom endoscopic scoring systems (Endoscopic grading of gastric intestinal metaplasia [EGGIM] and Kimura–Takemoto) were used; (3) proportion of patients with biopsies taken from at least two topographic sites (from both the antrum and the corpus, at the lesser and greater curvature of each); (4) proportion of patients with biopsies from the incisura; (5) proportion of patients with random biopsies (without a predefined location); (6) proportion of pathology reports using at least one system for histopathological staging (Operative link on gastritis assessment [OLGA]/Operative link on gastric intestinal metaplasia [OLGIM]); (7) proportion of pathology reports using subtyping of GIM as incomplete/complete (among those with GIM present on histology); (8) proportion of patients in whom *H. pylori* treatment was recommended (among those with *H. pylori* infection); and (9) proportion of patients with a recommendation for surveillance endoscopy mentioned in the clinical records (even if no further surveillance was advised).

### Statistical analysis

To assess changes in adherence to the MAPS guideline recommendations over time, we used descriptive statistics expressed as percentages. To quantify overall improvements in adherence across the time points, we additionally calculated the median adherence change per MAPS recommendation between 2010/2011 and both 2017/2018 and 2022/2023. These center-level changes were statistically evaluated using the Wilcoxon signed-rank test. The resulting median changes are presented with 95%CIs and corresponding *P* values, providing a robust summary of adherence trends over time. Notably, when interpreting these tests, it should be considered that recommendations with already high adherence levels in 2010/2011 had limited room for further improvement, potentially resulting in smaller overall adherence changes and wider confidence intervals.

A 10-percentage point threshold was used to define a substantial and practical shift in guideline adherence. An increase in adherence across the three time periods was defined as a minimum rise of 10 percentage points. This 10-percentage point absolute increase in adherence between time periods was defined a priori as a clinically relevant improvement, based on expert consensus and prior quality improvement literature in gastroenterology 12 13 . This threshold was chosen to reflect a change large enough to suggest potential systemic or behavioral shifts, rather than random variation.

For each recommendation, we aimed to perform a total of three comparisons (2017/2018 vs. 2010/2011 and 2022/2023 vs. 2010/2011) and a summary comparison (2017/2018 or 2022/2023 vs. 2010/2011) using the 10-percentage point cutoff. However, because certain guideline recommendations, such as targeted biopsies, virtual chromoendoscopy, and surveillance intervals, were only included in MAPS II and not in MAPS I, their impact can only be evaluated in the 2022/2023 period. If centers did not reach the full 100 patients per time period, analyses were conducted using the available sample size. Centers without data for a specific time point or recommendation were excluded from that analysis. In the results graphs, bars shown at 0% indicate data were available but there were no cases; absence of a bar and percentage figure indicates there were no data for that center/time point.

Adherence rates between academic and nonacademic centers, high and low volume hospitals, and MAPS guideline-developing countries were also compared. In addition to reporting statistical comparisons, we focused on the magnitude and direction of change and the accompanying confidence intervals, to allow readers to assess both statistical and clinical relevance. All analyses were performed using SPSS version 28.

### Results

A total of nine endoscopy centers across seven countries were included in the analysis. The number of patients varied per center and time period, with a total of 2426 patients included in the three time periods: 2010/2011, 2017/2018, and 2022/2023 ( Table 1 ). The number of patients included per time period was 583 patients in 2010/2011, 878 in 2017/2018, and 965 in 2022/2023. The number of patients included from each center differed across the time periods. Colentina Clinical Hospital (Romania) and Connolly Hospital Blanchardstown (Ireland) provided data solely for the 2017/2018 and 2022/2023 periods, with 100 patients included per period. Ospedale Universitario Sant'Andrea (Italy), Unidade Local de Saúde de Santo António (Portugal), Maasstad Hospital (The Netherlands), Erasmus Medical Center (The Netherlands), and Hospital Clínic de Barcelona (Spain) reported consistent patient numbers (100 each) across all three periods. John Radcliffe Hospital (UK) showed an increase in sample size during the later years (2017/2018 and 2022/2023), and Coimbra, Unidade Local de Saúde de Coimbra (Portugal) included 50 patients per period.

**Fig. 1:**
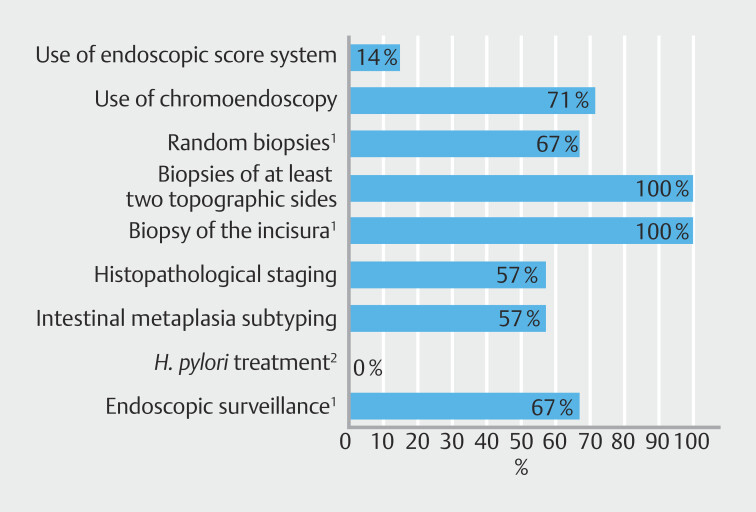
Overall proportion of centers that achieved at least a 10-percentage point increase in adherence for each recommendation in 2022/2023 or 2027/2018 vs. 2010/2011 (n = 7). ^1^ n = 6 for random biopsies, biopsy of the incisura, and endoscopic surveillance. ^2^ No improvement was needed.

Based on the available data from several centers, annual upper GI endoscopy volumes were estimated to range between 1500 and 20000 procedures, with approximately 50–2000 patients diagnosed with GIM per study period. Over the years, most centers showed improvement in seven of the nine recommendations ( Fig. 1 ). The improvement of the centers for each recommendation across the three time points is presented in **Table 1s** , see online-only Supplementary material. Adherence did not differ between academic and nonacademic centers or high and low volume hospitals, but was higher in MAPS guideline-developing centers (Portugal, Italy, The Netherlands).

### Upper GI endoscopy procedure and biopsy practices

**Fig. 2:**
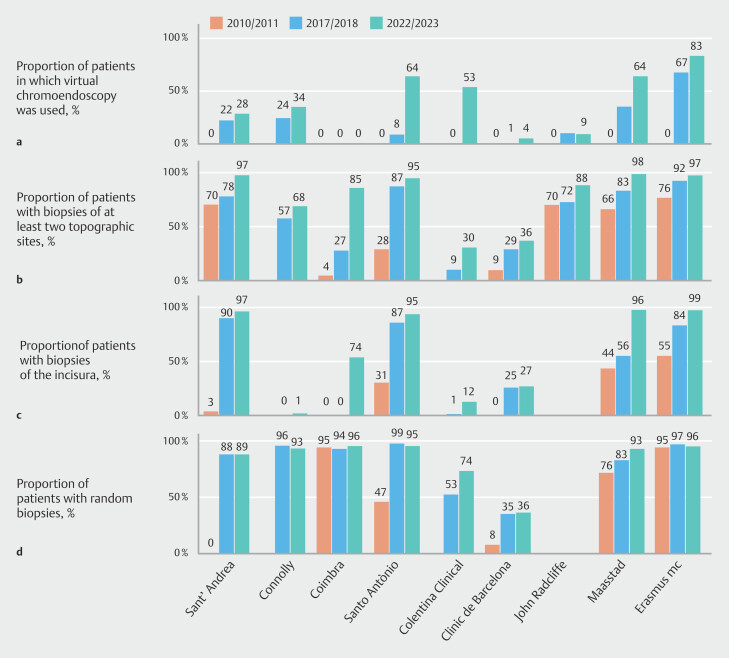
Proportions of patients in each center and period evaluated: **a** in whom virtual chromoendoscopy was used; **b** with biopsies from at least two topographic sites in the stomach ^1^ ; **c** with biopsies of the incisura; **d** in whom random biopsies were collected ^2^ . ^1^ From both the antrum and the corpus, at the lesser and greater curvature of each. ^2^ Among those with endoscopic biopsies.

Six units improved in virtual chromoendoscopy adherence, and almost all centers improved significantly in adherence to biopsy protocols. In 2010/2011, chromoendoscopy was not routinely used in any center. By 2017/2018, its use had increased, reaching 34% or more in two centers, while five remained below 10%. By 2022/2023, six centers showed increased usage, with rates ranging from 28% to 83%, while three centers still had minimal or no use (9%, 4%, and 0%) ( Fig. 2 **a** ). The median overall increase was 28% (95%CI 2% to 80%; *P* = 0.003). Endoscopic staging scores were used in almost none of the centers during any of the study periods.

All centers improved significantly in performing biopsies from the two compartments (antrum and corpus), with adherence improving from 4%–76% in 2010/2011 to 9%–92% in 2017/2018, and further to 30%–99% by 2022/2023, with an overall median increase of 27% (95%CI 18% to 79%; *P* = 0.006) ( Fig. 2 **b** ). Biopsies from the incisura increased from a range of 0%–55% in 2010/2011 to 0%–90% in 2017/2018, and further to 0%–99% by 2022/2023, with four centers exceeding 90% ( Fig. 2 **c** ). The median overall increase was 54% (95%CI 29% to 90%; *P* = 0.002), with adherence improving in five centers by 2017/2018 and in seven centers by 2022/2023. The proportion of patients with random biopsies taken during endoscopy also increased over time, with an overall median increase of 20% (95%CI 5% to 46%; *P* = 0.04). Four centers showed an increase from 0%–95% in 2010/2011 to 35%–99% in 2017/2018, and 36%–96% by 2022/2023, with five centers exceeding 90% ( Fig. 2 **d** ).

### Histopathology staging and subtyping

**Fig. 3:**
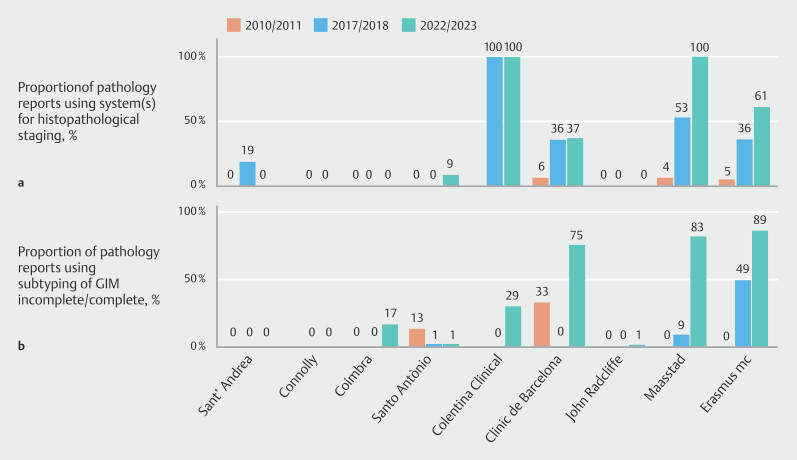
Proportion of pathology reports in each center and period evaluated using: **a** at least one system for pathological staging; **b** subtyping of gastric intestinal metaplasia (GIM) as incomplete/complete among those with GIM reported on histology.

Improvement in the use of histological scores and the diagnosis of complete or incomplete intestinal metaplasia (IM) was reported in five units ( Fig. 3 ). Histopathological staging was minimally used in 2010/2011, with percentages ranging from 0%–6%. By 2017/2018, five centers increased their usage to 19%–100% and, in 2022/2023, this practice continued to increase in five centers, with percentages ranging from 8%–100%. Although the other four centers remained at 0%, the median overall increase was 31% (95%CI 0% to 90%; *P* = 0.09).

The subtyping of IM into complete and incomplete GIM remained low, with most of the centers being at 0% in the periods 2010/2011 and 2017/2018, with only one center at 49%. By 2022/2023, this had increased in five centers with percentages ranging from 17% to 86%, although the other four centers remained below 1% ( Fig. 3 **b** ). The overall median increase was 23% (95%CI 10% to 85%; *P* = 0.08).

### Helicobacter pylori treatment and surveillance endoscopy

**Fig. 4:**
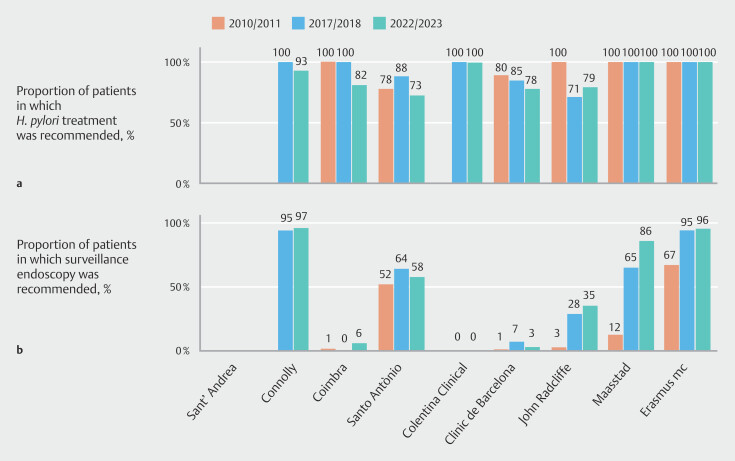
Proportion of patients in each center and period evaluated: **a** in whom *H. pylori* treatment was recommended ^1^ ; **b** with a recommendation for surveillance endoscopy mentioned in their clinical records ^2^ . ^1^ Among those with *H. pylori* infection. ^2^ Mentioned, even if no further surveillance was advised.

Recommendations regarding *H. pylori* treatment were consistently high across all time periods, whereas surveillance endoscopy recommendations showed improvement in four centers ( Fig. 4 ). *H. pylori* treatment recommendations were 78%–100% of centers recommending treatment in 2010/2011, 71%–100% in 2017/2018, and 73%–100% in 2022/2023, with a median change of −5% (95%CI −18% to 0%; *P* = 0.07).

Recommendations for surveillance endoscopy showed gradual improvement over time, rising from 0%–67% in 2010/2011 to 7%–95% in 2017/2018, and remaining similar in 2022/2023 at 3%–97%. The overall increase was 18% (95%CI 3% to 67%; *P* = 0.06). Despite this increase, adherence remained suboptimal, with surveillance endoscopy being mentioned in the clinical report of fewer than 35% of patients in half of the centers (4/8) in 2022/2023 ( Fig. 4 **b** ).

## Discussion

This study evaluated the adherence to the MAPS I and II guideline recommendations for managing premalignant gastric conditions across various European centers over three time periods (2010/2011, 2017/2018, and 2022/2023). While most centers showed improvements in adherence to the guideline recommendations, several aspects remained suboptimal, highlighting important gaps with potential clinical impact, particularly in terms of timely detection and risk stratification of gastric neoplastic conditions. Notably, adherence varied across centers and differed by recommendation. This variability was also reflected in the relatively wide confidence intervals of the center-level adherence changes per recommendation.

Unlike previous studies, we found no clear difference in adherence to the guidelines between academic/nonacademic or high/low volume hospitals, suggesting that clinician knowledge, risk awareness, and institutional factors may play a larger role than hospital status 14 ; however, centers involved in developing the MAPS guidelines generally showed better adherence (Portugal, Italy, The Netherlands), likely owing to their deeper understanding of the guidelines and more consistent implementation of standardized protocols. In contrast, centers outside this group may lack awareness and resources, causing variability in adherence. Adherence to the MAPS recommendations may also be influenced by the setting in which endoscopy is being performed. Endoscopists in dedicated surveillance units may be more familiar with and motivated to follow guidelines and more likely to document surveillance in the clinical report than those in general dyspepsia clinics, where diagnostic priorities may differ.

Our data were collected at center level, without accounting for interendoscopist variability. Differences between experienced endoscopists and trainees could introduce internal inconsistencies. Additionally, whether an upper GI endoscopy protocol was used consistently across all time points for each center was not retrievable. Nonetheless, better coordination between endoscopists and pathologists, such as explicit requests to apply OLGA/OLGIM scoring in histopathology reports, could improve adherence to standardized risk stratification. Additionally, standardized reporting, a key quality parameter within the ESGE quality initiative, would further enhance consistency in assessments and strengthen the validity of retrospective data analysis. Addressing these gaps through targeted education and institutional support is crucial for improving compliance and ensuring high quality care across all centers 15 16 .

All nine centers adhered to the MAPS guideline for *H. pylori* treatment, and consistently demonstrated high adherence rates across all time periods. The rates were already high in the pre-MAPS period, indicating that *H. pylori* eradication is regarded as an established strategy for gastric cancer prevention 7 .

Nevertheless, several recurring barriers to adherence could be pinpointed, affecting various aspects of guideline implementation. The limited application of virtual chromoendoscopy, as well as endoscopic and histopathological staging systems, can be attributed to several factors, including high costs, the need for specialized training, and the perception that these techniques are more time-consuming than standard methods 17 18 . Although narrow-band imaging (NBI) has demonstrated superior accuracy in detecting IM, with minimal additional time investment and improved cost-effectiveness, its adoption remains inconsistent 19 20 21 . Similarly, promising endoscopic staging systems such as the Kimura–Takemoto and EGGIM systems remain underutilized. The limited use of the Kimura–Takemoto system may be partly explained by regional disparities: while it is widely used in some East Asian countries, it is less common in other parts of the world 22 . The EGGIM score, introduced in 2016, is still relatively new, which may explain the limited familiarity with this staging system 20 23 . These findings suggest that targeted educational initiatives and increased resource allocation could facilitate the broader adoption of these advanced endoscopic and histopathological techniques.

While overall biopsy protocol adherence has improved, challenges remain in standardization and documentation. Our results are consistent with, but slightly higher than, studies from 2023 and 2021 across three and 10 European centers, respectively 10 24 . In the 2021 study, biopsies were taken in 56.6% of cases, with higher rates in MAPS-adherent centers (68.1%) than in those following the Sydney classification (46.0%) 24 . The second study reported a 68% compliance rate for random biopsies 10 ; notably, in 8% of cases, biopsy location was undetermined. In our study, adherence to random biopsies increased in four centers, and adherence to incisura biopsies improved in six centers, with four centers achieving rates exceeding 90% by 2022/2023. This aligns with a 2018 Italian survey that also reported a 90% adherence rate to the MAPS biopsy protocol 9 . Additionally, adherence to obtaining biopsies from two sites improved in seven centers. Although high adherence to the biopsy protocols was observed, nonadherance in biopsy handling persisted, with some samples from different gastric regions being pooled together, instead of being placed in separate containers.

The location of GIM is crucial for surveillance recommendations, as it directly influences risk assessment. Additionally, GIM subtyping is of particular importance, as the incomplete subtype has been associated with a higher risk of progression to gastric cancer. Subtyping was successfully implemented, showing notable improvement in five centers; however, in the remaining four centers, its usage remained below 1%. These gaps are more likely due to a lack of routine endoscopic and pathological implementation, rather than financial or training constraints, as research indicates that subtyping is relatively simple, requires minimal cost and effort, and is well within the capabilities of trained pathologists 25 26 27 .

Surveillance endoscopy, critical for early gastric cancer detection, increased in four out of seven centers, but adherence rates stayed suboptimal. Similar trends were seen in a study from the UK and the Netherlands, which showed that adequate surveillance was applied in only 54.3% of patients with GIM or gastric atrophy 10 . Adherence to follow-up guidelines may be hindered by several factors, including poor communication about the importance of surveillance, limited access to endoscopic services, and patient-related barriers such as noncompliance or logistical challenges. Additionally, it is still unclear whether gastroenterologists consistently understand and apply the recommended surveillance intervals. Awareness of key gastric cancer risk factors – such as country of origin, autoimmune gastritis, and family history – may also be lacking, potentially leading to suboptimal risk stratification and surveillance recommendations.

To our knowledge, this is the first study to evaluate adherence to the recommendations included in the MAPS guidelines, covering both histopathological and upper GI endoscopic protocols. Although our study focuses on MAPS guideline adherence, rather than treatment outcomes for the patients, the existing literature clearly shows that following endoscopic and histopathological protocols improves early detection of preneoplastic conditions, ultimately reducing gastric cancer mortality 10 25 . The observed improvements in adherence are a crucial step toward better healthcare, though the direct clinical impact requires further investigation.

Several limitations to our study should be noted. Variability in data availability and sample sizes may have influenced our analysis. Additionally, two centers lacked data for 2010/2011 and, although they demonstrated high adherence rates during the other two periods, they could not be included in the analysis comparing pre- and post-MAPS. Moreover, self-reported adherence could have resulted in either an over- or underestimation. Potential variability in endoscopist practices within centers may also have influenced adherence, differing by indication, with surveillance endoscopists likely more trained and motivated than those in dyspepsia settings.

The nine included centers may not fully represent European healthcare settings – one of the centers for example was located in Eastern Europe – which may limit generalizability. Our findings might not fully reflect adherence behaviors or clinical challenges in areas with different disease prevalences or healthcare infrastructures. However, the inclusion of centers with similar findings despite having lower prevalences of gastric precancerous conditions suggests that prevalence may not play a significant role in adherence to guidelines. Differences in data collection methods and unmeasured factors, such as healthcare policies and patient demographics, may also have affected adherence levels.

Although guideline adherence is the focus of this study, it is important to acknowledge that guidelines must be not only followed, but also applied appropriately. This study does not evaluate the adequacy or clinical effectiveness of adherence. Future research should aim to assess whether adherence leads to improved clinical outcomes, such as earlier detection of gastric cancer or improved survival rates. Notably, the relatively low rate of centers willing to participate in this study (42.9%) may have introduced selection bias, as centers that agreed to participate might be more motivated to collect data or be more familiar with guideline implementation. This may also suggest that, in those centers not participating or other centers not included, some of the results may be worse, which fundamentally supports the need for continuous educational efforts.

Moreover, although improvements over time align with the MAPS publication dates, causality cannot be confirmed. Other factors, such as national policy changes, local quality initiatives, or the availability of technology, could also explain the observed changes.

Despite these limitations, our study highlights significant variability in adherence and names key areas for intervention to improve compliance and enhance gastric cancer prevention. In conclusion, while notable improvements in MAPS guideline adherence were seen across all centers and countries, substantial variation remains. Key challenges persist in surveillance endoscopy and histopathological staging, both of which are crucial for early detection.

Addressing local barriers through targeted education, standardization of diagnostic protocols, integrating MAPS guidelines into electronic reporting forms, and improved clinician awareness are critical for enhancing compliance. Further studies should include a broader range of centers across more diverse regions and focus on the clinical effectiveness of adherence and strategies to improve early detection and risk stratification, ultimately advancing the quality of healthcare for gastric cancer prevention.

## Supplementary Material

Supplementary Material Zusatzmaterial
