# Supplementary material for: Adherence to clinical practice guidelines for Management of epithelial precancerous conditions and lesions in the stomach in Europe
Source: Endoscopy. 2025 Oct 6;57(12):1338–47. doi: 10.1055/a-2695-1376 (PMC12668271; doi:10.1055/a-2695-1376)
Supplement: Supplementary file 1 — Supplementary Material [file 10-1055-a-2695-1376_27047401.pdf]

Supplementary material

Adherence to clinical practice guidelines for management of epithelial precancerous conditions and lesions in the stomach in nine European centers

Filipa Fontes, Noa E.A. Kapteijn, Cesare Hassan, Charlene Deane, Margarida Cristiano, Henrique Fernandes-Mendes, Irina Luzko, Maša Čavlina Sevo, Orlaith Kelly, Gianluca Esposito, I. Lisanne Holster, Michiel P. J. van der Horst, Charlotte F. Kweldam, Andrei Voiosu, Ricardo Marcos-Pinto, Nuno Almeida, Colm O'Morain, Leticia Moreira, Jan Bornschein, Manon C.W. Spaander, Mário-Dinis-Ribeiro

Table 1s. Number of at least 10% improved centers across the three time points.

MAPS recommendation	2017/2018 vs. 2010/2011 (N=7)	2022/2023 vs. 2017/2018 (N=9)	2022/2023 or 2017/2018 vs. 2010/2011 (N=7)
Use of chromoendoscopy	4	5	5
Use of endoscopic score system	0	1	1
Biopsies of at least two topographic sides	5	6	7
Biopsy of the incisura	5	4**	6*
Random biopsies	4	2**	4*
Histopathological staging	4	2	4
IM subtyping	1	5	4
H. pylori treatment[1]	0	0	0
Endoscopic surveillance	4	1	4*

[1] No need for improvement
*N=6
**N=8
